# Supplementary material for: Silica-rich volcanism in the early solar system dated at 4.565 Ga
Source: Nat Commun. 2018 Aug 2;9:3036. doi: 10.1038/s41467-018-05501-0 (PMC6072707; doi:10.1038/s41467-018-05501-0)
Supplement: Supplementary file 1 — Supplementary Information [file 41467_2018_5501_MOESM1_ESM.pdf]

Supplementary Information

Title: Silica-Rich Volcanism in the Early Solar System Dated at 4.565 Ga

Srinivasan et al.

Supplementary Methods

Fe-Mg Mineral-Melt Exchange Coefficients

Calculations for OPX-Melt, CPX-Melt, and OPX-CPX Mg-Fe exchange coefficients are given in Supplementary Data 4, and correspond with areas labeled in Supplementary Figure 6. FeO and MgO values for two enstatite phenocrysts were combined to obtain an average OPX FeO and MgO value. FeO and MgO values for four augite phenocrysts were combined to obtain an average CPX FeO and MgO value. The six matrix maps obtained through X-ray mapping were additionally combined to obtain an average FeO and MgO groundmass value. Fe and Mg distribution coefficients were calculated first using the equation:

$$D_{Fe} = \frac{X_{Fe}^{OPX}}{X_{Fe}^{Melt}} \qquad (1)$$

where X is the concentration of FeO in weight percent. The orthopyroxene phenocrysts exhibit melt-pyroxene D_{Fe} ratio of 1.25 ± 0.20 and D_{Mg} ratio 5.02 ± 0.51. The clinopyroxene phenocrysts exhibit melt-pyroxene D_{Fe} ratio of 0.68 ± 0.13 and D_{Mg} ratio 3.12 ± 0.51.

The Fe-Mg exchange coefficient was calculated using the following equation:

$$K_d^{Fe-Mg} = \frac{D_{Fe}}{D_{Mg}} \qquad (2)$$

The orthopyroxene and clinopyroxene phenocrysts exhibit melt-pyroxene Mg-Fe exchange K_D ratios of 0.25 ± 0.05 and 0.22 ± 0.06. The orthopyroxene and clinopyroxene phenocrysts exhibit an OPX-CPX Mg-Fe exchange K_D ratio of 0.88 ± 0.10.

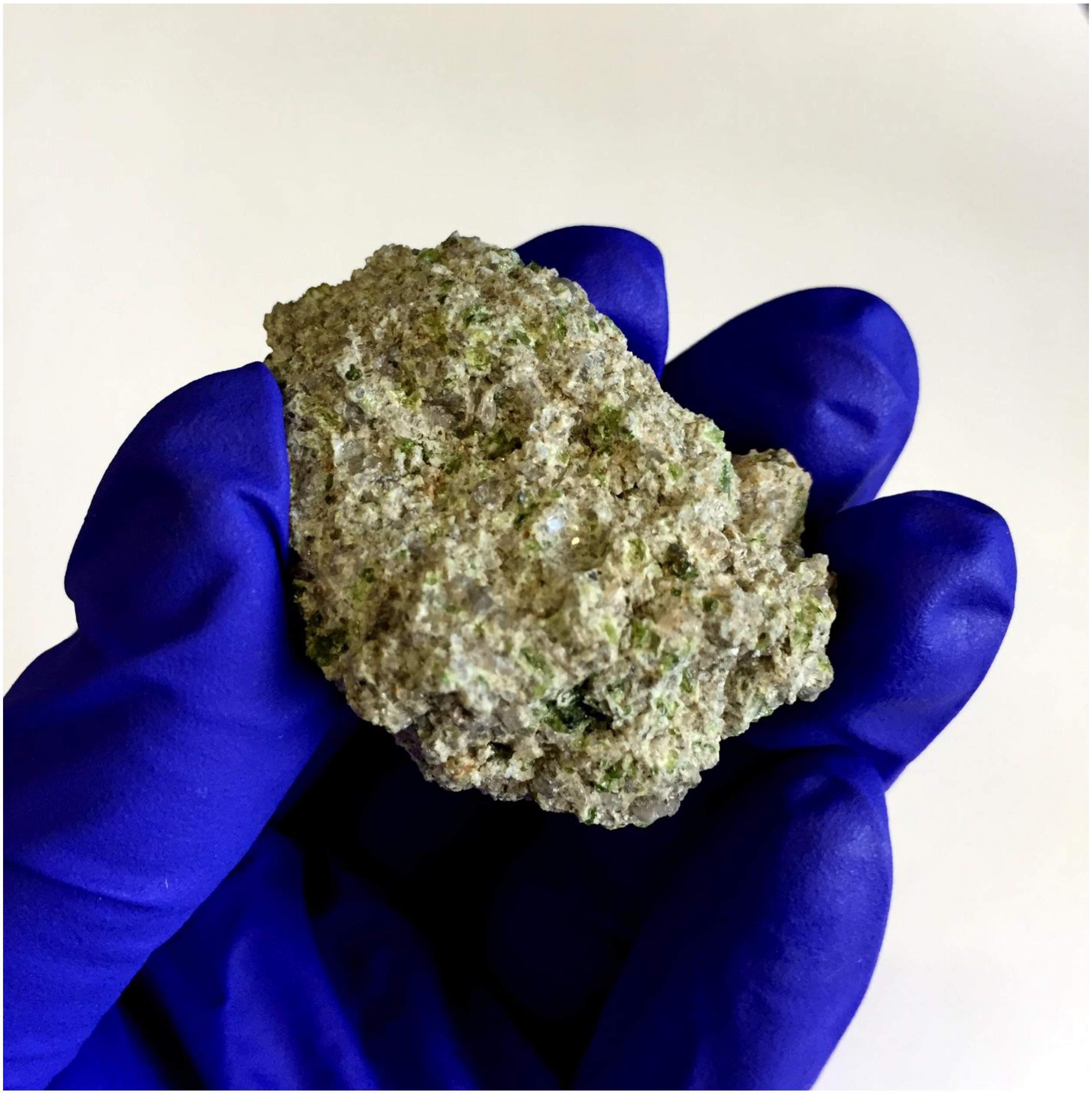

Supplementary Figure 1. Photograph of the NWA 11119 deposit sample at the Institute of Meteoritics (UNM). Green is Cr-rich pyroxene, grey is silica polymorph, and white is plagioclase. Note spherical cavity in the center of the image.

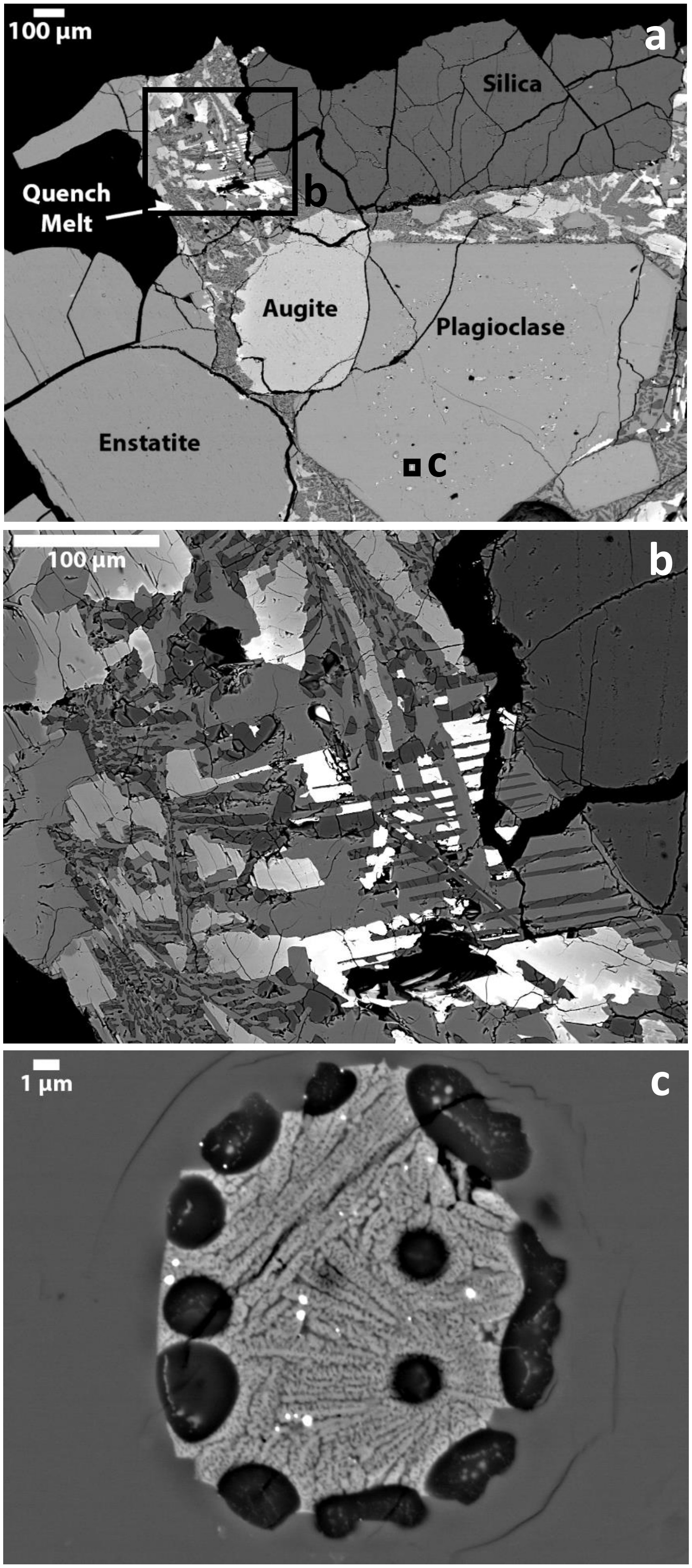

Supplementary Figure 2. a) Backscattered electron (BSE) image of the mm-sized phenocrysts (silica, augite, enstatite, and plagioclase) and the surrounding quench melt in NWA 11119. b) Enlarged image of (a) showing quench melt. c) Enlarged image of (a) showing an inclusion of quenched inclusions within a plagioclase phenocryst.

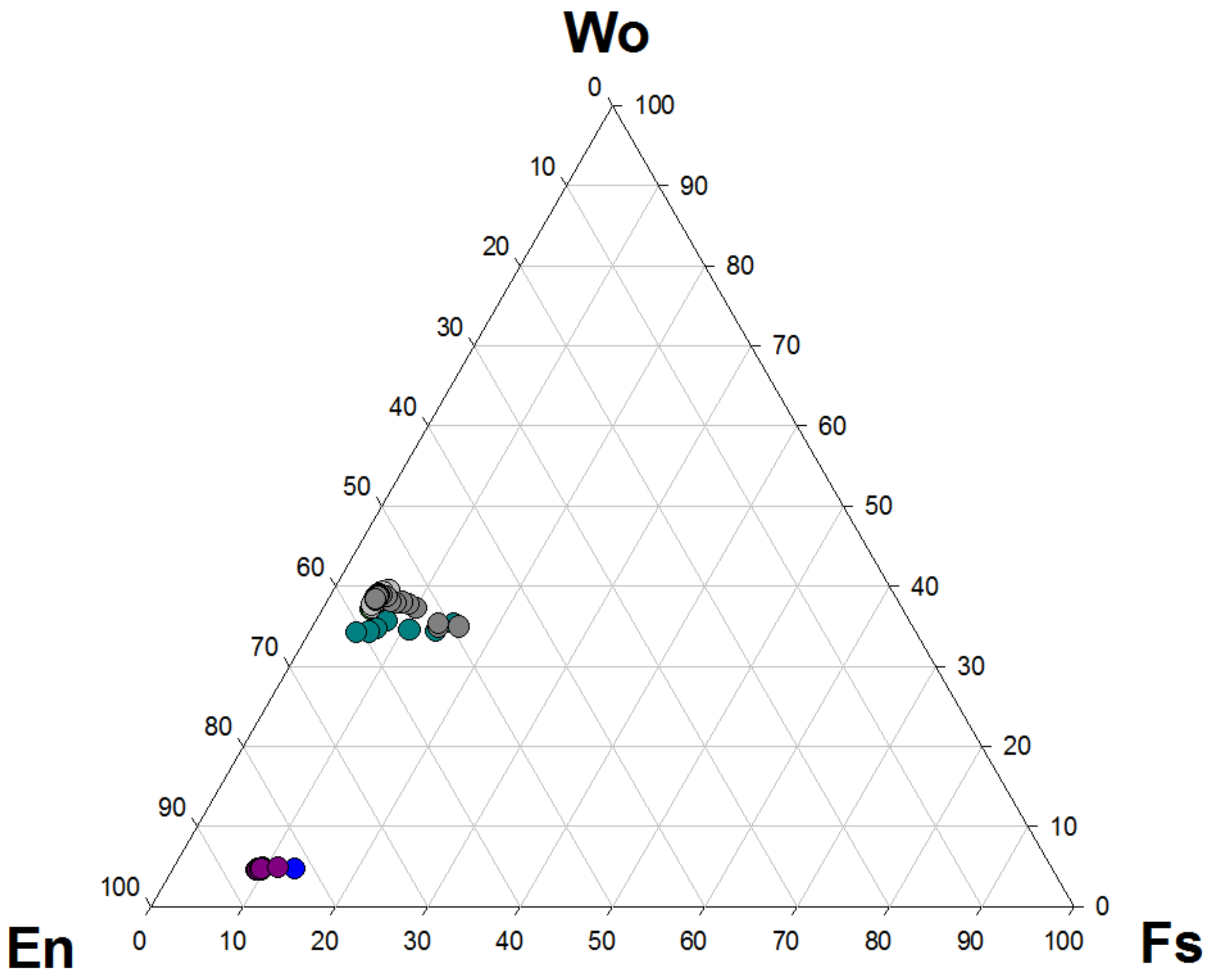

Supplementary Figure 3. Pyroxene ternary diagram plotting line arrays from phenocrysts. NWA 11119 contains two types of pyroxene phenocrysts: high-Ca augite and low-Ca enstatite.

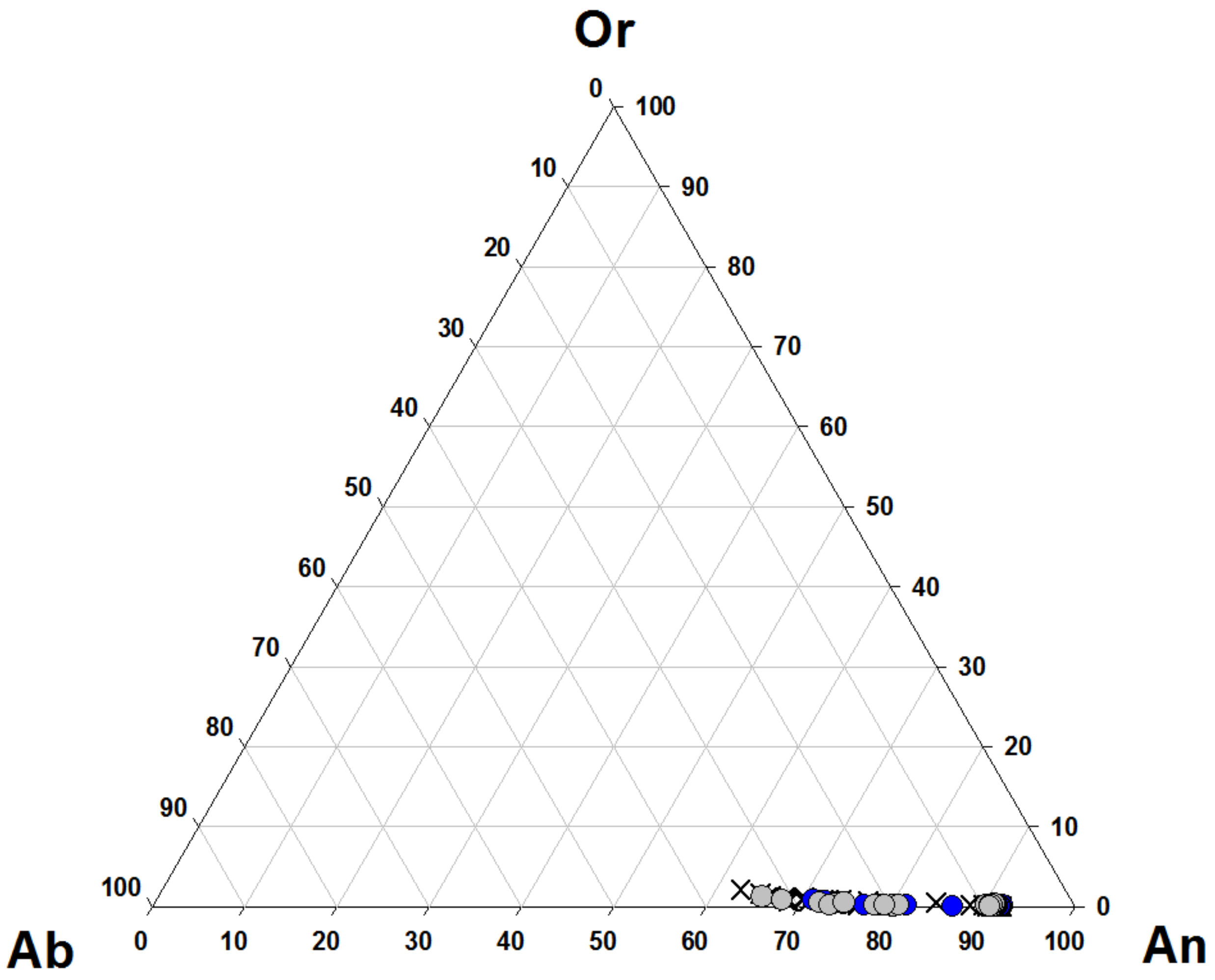

Supplementary Figure 4. Feldspar ternary diagram plotting line arrays from feldspar phenocrysts (circles) and spot analyses from feldspar matrix grains (crosses).

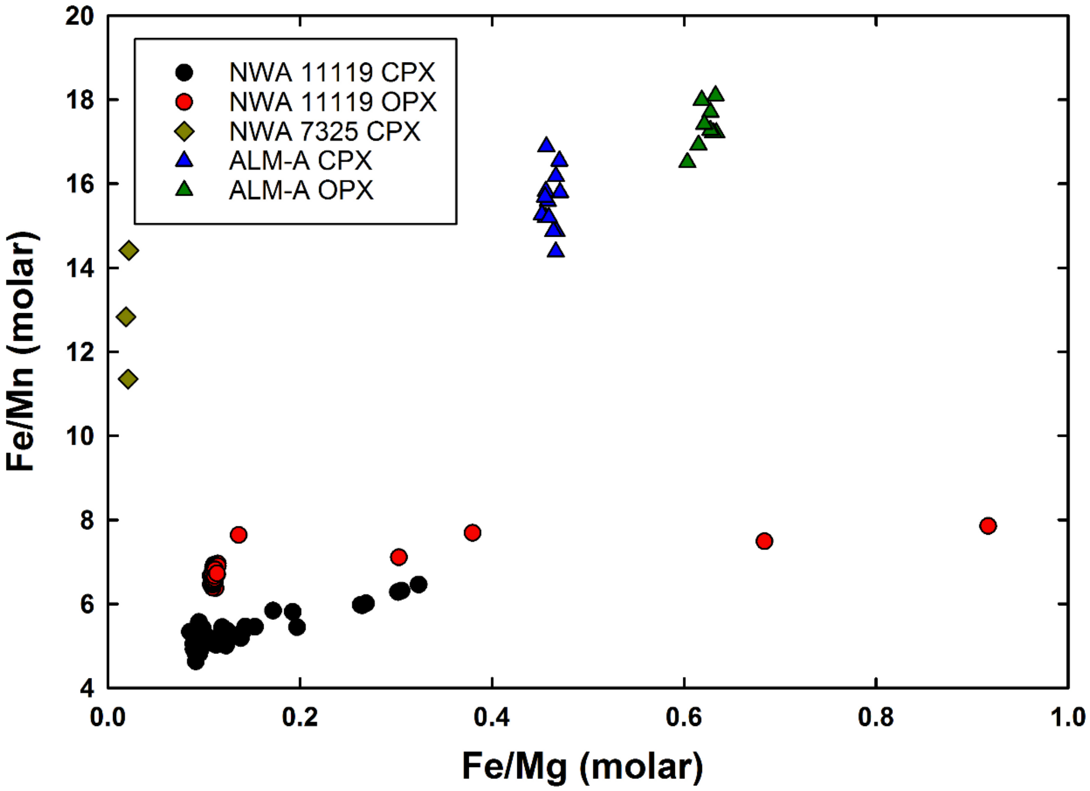

Supplementary Figure 5. Molar Fe/Mn ratios of pyroxenes plotted against pyroxene molar Fe/Mg. Data for NWA 11119 are from the present study. Data from ALM-A are from *(1)*, and data from NWA 7325 are from *(2-4)*.

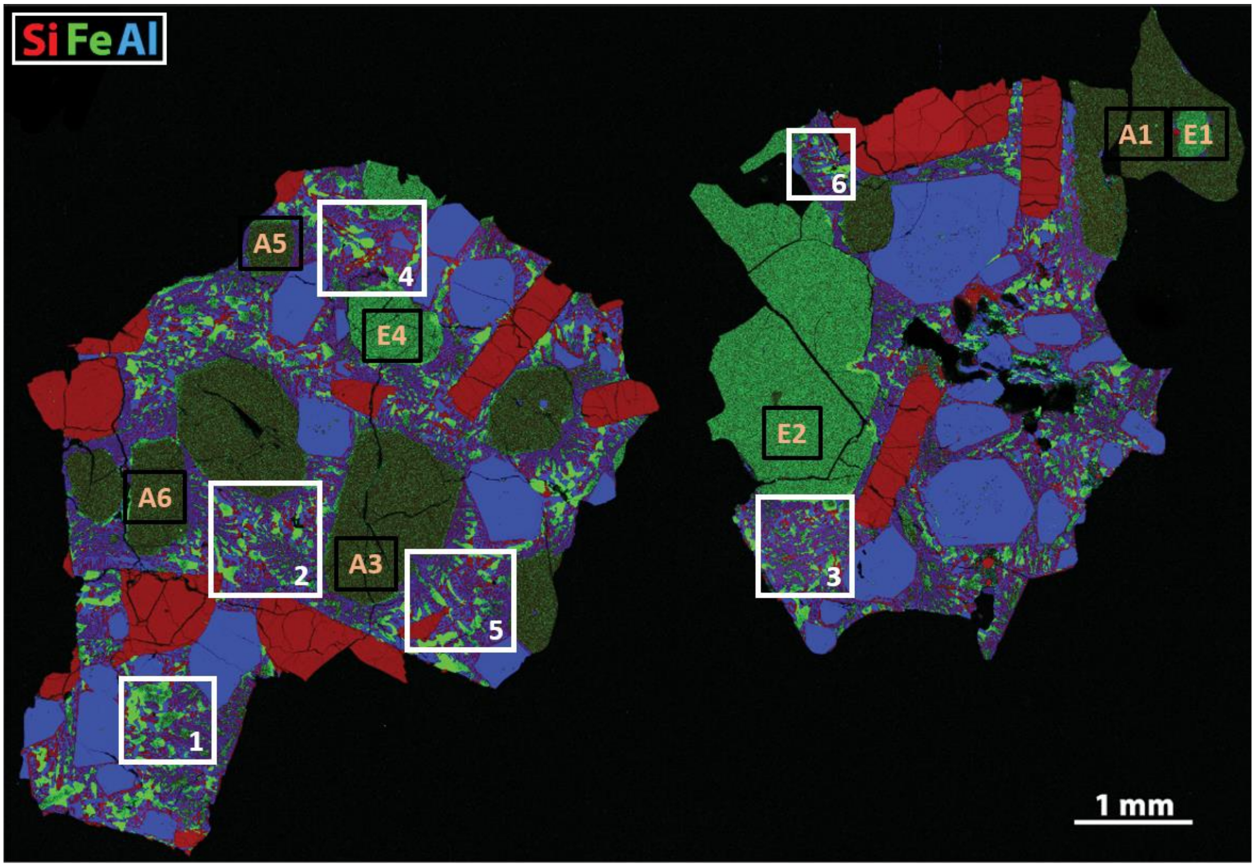

Supplementary Figure 6. Labeled false-colored X-ray mosaic of the NWA 11119 slice. Line arrays from pyroxene phenocrysts (E=enstatite, A=Augite) that were used to calculate K_D ratios are indicated as black boxes. Groundmass areas mapped by quantitative EPMA techniques are indicated as white boxes. This figure corresponds with Supplementary Data 2 and 4.

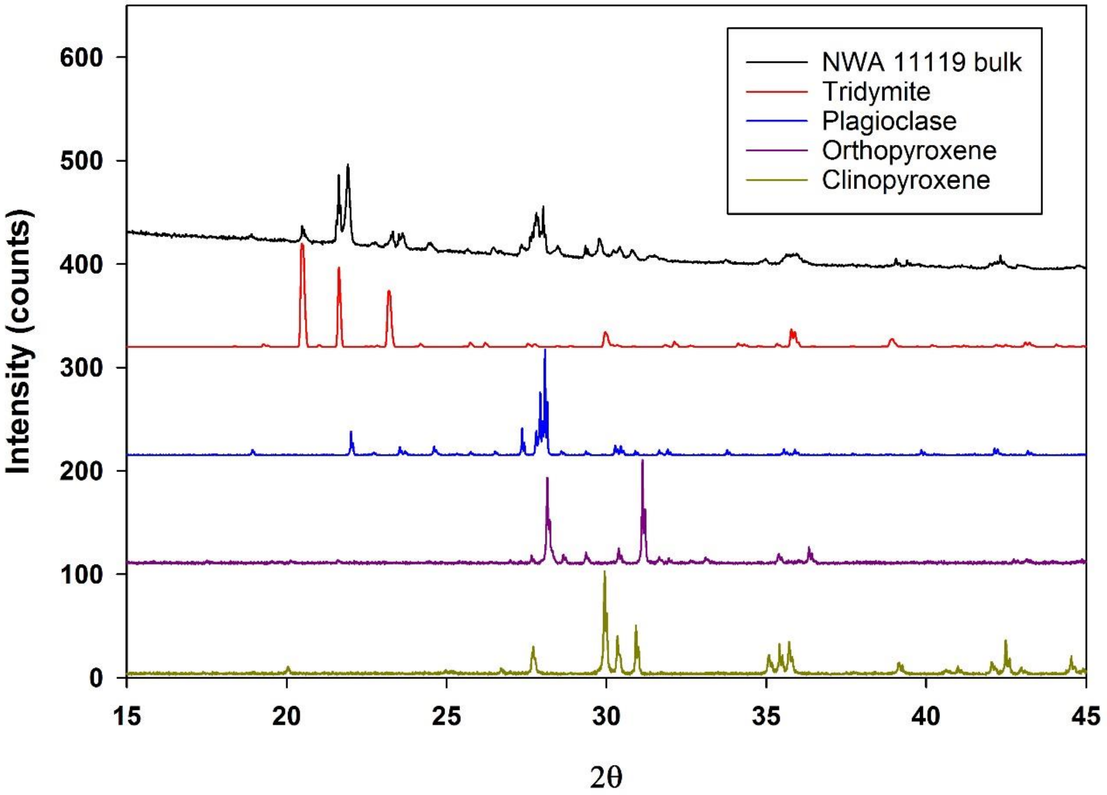

Supplementary Figure 7. X-ray diffraction (XRD) patterns of whole-rock powdered sample of NWA 11119. XRD patterns of each of the major phases that comprise NWA 11119 are also provided for comparison to account for components that make up the bulk rock pattern for NWA 11119. The XRD data for tridymite (R090042), plagioclase (R040059), orthopyroxene (R040093), and clinopyroxene (R040097) are from the RRUFF database (http://rruff.info/)

Supplementary References

1. Bischoff, A. et al. Trachyandesitic volcanism in the early Solar System. *Proc. Natl. Acad. Sci.* **111,** 12689-12692 (2014).
2. Goodrich, C. A. et al. Petrogenesis and provenance of ungrouped achondrite Northwest Africa 7325 from petrology, trace elements, oxygen, chromium and titanium isotopes, and mid-IR spectroscopy. *Geochim. Cosmochim. Acta* **203,** 381-403 (2017).
3. Barrat, J. A. et al. Crustal differentiation in the early solar system: Clues from the unique achondrite Northwest Africa 7325 (NWA 7325). *Geochim. Cosmochim. Acta* **168,** 280-292 (2015).
4. Weber, I. et al. Cosmochemical and spectroscopic properties of Northwest Africa 7325—A constorium study. *Meteorit. Planet. Sci.* **51,** 3-30 (2016).
