# Supplementary material for: Silica-rich volcanism in the early solar system dated at 4.565 Ga
Source: Nat Commun. 2018 Aug 2;9:3036. doi: 10.1038/s41467-018-05501-0 (PMC6072707; doi:10.1038/s41467-018-05501-0)
Supplement: Supplementary file 3 — Description of Additional Supplementary Files [file 41467_2018_5501_MOESM3_ESM.pdf]

Description of Additional Supplementary Files

File Name: Supplementary Movie 1
Description: A 360° CT scan of the vesicle/cavity content in the NWA 11119 deposit sample, which we have determined to be 1.2 vol.%.

File Name: Supplementary Movie 2
Description: A CT scan video segmenting the Z-axis of the deposit sample showing the differences in mineralogy of NWA 11119 (color legend: silica in red, pyroxene in yellow, plagioclase in blue, oxides in white).

File Name: Supplementary Data 1
Description: Pyroxene, feldspar, and silica phenocryst compositions by EPMA.

File Name: Supplementary Data 2
Description: Calculated MgFe exchange coefficients.

File Name: Supplementary Data 3
Description: Matrix compositions by EPMA.

File Name: Supplementary Data 4
Description: Calculated bulk rock and matrix compositions for NWA 11119.

File Name: Supplementary Data 5
Description: Major, minor and rare earth element (REE) concentrations (measured by ICPMS) for a whole-rock (WR) fraction and mineral separates from NWA 11119.

File Name: Supplementary Data 6
Description: Oxygen isotope ratios for NWA 11119.

File Name: Supplementary Data 7
Description: 27Al/24Mg ratios and Mg isotope data for the whole-rock fraction and mineral separates of NWA 11119.
